# What is pH regulation, and why do cancer cells need it?

**DOI:** 10.1007/s10555-018-09778-x

**Published:** 2019-02-01

**Authors:** Pawel Swietach

**Affiliations:** Department of Physiology, Anatomy and Genetics, Parks Road, Oxford, OX1 3PT England

**Keywords:** Tumors, Active transport, Carbonic anhydrase, Monocarboxylate transport, Set point, Lactate

## Abstract

Metabolism is a continuous source of acids. To keep up with a desired metabolic rate, tumors must establish an adequate means of clearing their acidic end-products. This homeostatic priority is achieved by various buffers, enzymes, and transporters connected through the common denominator of H^+^ ions. Whilst this complexity is proportionate to the importance of adequate pH control, it is problematic for developing an intuition for tracking the route taken by acids, assessing the relative importance of various acid-handling proteins, and predicting the outcomes of pharmacological inhibition or genetic alteration. Here, with the help of a simplified mathematical framework, the genesis of cancer pH regulation is explained in terms of the obstacles to efficient acid venting and how these are overcome by specific molecules, often associated with cancer. Ultimately, the pH regulatory apparatus in tumors must (i) provide adequate lactic acid permeability through membranes, (ii) facilitate CO_2_/HCO_3_^−^/H^+^ diffusivity across the interstitium, (iii) invest in a form of active transport that strikes a favorable balance between intracellular pH and intracellular lactate retention under the energetic constraints of a cell, and (iv) enable the necessary feedback to complete the homeostatic loop. A more informed and quantitative approach to understanding acid-handling in cancer is mandatory for identifying vulnerabilities, which could be exploited as therapeutic targets.

## Introduction

Tissue compartments will invariably contain H^+^ ions from the ionization of water and a myriad of biochemical substances. The concentration of these ions, commonly expressed on a pH scale [1], influences the activity of all proteins that undergo protonation: the most rapid and reversible post-translational modification [2–4]. The activity of enzymes, for instance, is strongly influenced by changes in pH, which is one reason why certain types of enzymes are grouped together in sub-cellular compartments of distinct pH, such as proteolytic enzymes inside acidic lysosomes [5]. A collection of enzymes can be ascribed an optimal pH; for example, the ensemble of cytoplasmic enzymes, including those involved in glycolysis, is predicted to operate optimally near pH 7.3 [5], and it should be in the interest of cells to maintain cytoplasmic pH near to this level.

If there was no net production of acids (or bases) in cells, tissue pH could remain constant, even in the absence of a dedicated regulatory system. However, essentially all tissues, including tumors, are net-producers of acid because mitochondrial respiration and fermentative metabolism generate large flows of CO_2_ and lactic acid, respectively [6, 7]. Genetic and epigenetic changes [8], as well as oxygen depletion, reprogram cancer metabolism towards a more glycolytic phenotype [9], but in order to adequately supply ATP, this low-yielding energy pipeline must be upregulated, resulting in an exacerbated output of lactic acid [6, 10]. Aberrant blood perfusion, which is a characteristic of many tumors, erects a barrier to the efficient venting of this acidic burden [11, 12]. A consequence of these circumstances is low extracellular pH (pH_e_), a chemical signature of the tumor microenvironment [13–16].

Microenvironmental acidity is not merely a collateral waste product of tumor biology, but a valuable source of feedback that controls various processes [17–20], including metabolic rate [21]. The sum of the effects of pH on cell biology is powerful enough to influence survival, which has been likened to a selection process favoring a particular phenotype of cancer cell among a genetically diverse population [6, 22, 23]. In order for acid-driven somatic evolution to take place, there must be a means by which the successful (and presumably more aggressive) subpopulations have adapted to microenvironmental acidity. Such a survival advantage can take one of two forms, which are not mutually exclusive.

The first involves a re-modeling of pH sensitivity, which could be achieved through genetic mutations involving titratable residues, such as histidines [4, 24–26]. The protonation state of histidine changes dramatically over the expanded physiological range, bestowing proteins with exquisite pH-dependence [27–29]. A shift in the pH sensitivity curve may, for example, allow mutant proteins to remain active even at an abnormal level of pH [4]. The scope of this effect on cell biology is, however, restricted to the functional remit of the mutated protein.

Since a large fraction of pH-sensitive proteins resides inside cells, another adaptation to an acidic microenvironment is for cells to defend a favorable (usually alkaline) intracellular pH (pH_i_), using an appropriately powered homeostatic mechanism. This adaptive strategy has the advantage of influencing all intracellular proteins collectively. A “perfect” homeostatic system would keep the pH of the internal environment constant at the set point, irrespective of the external conditions or other constraints; in achieving this, cells acquire a substantial degree of independence, which is particularly empowering for cancer cells. However, cells placed under acid stress will not universally manifest such perfect pH_i_ homeostasis; instead, there will be variation in regulatory prowess which relates to “acid fitness” and could provide substrate for selection pressures. pH-regulatory proteins underpin this phenotype, and in recent years, much attention has been given to testing their therapeutic utility [30–33].

**Fig. 1:**
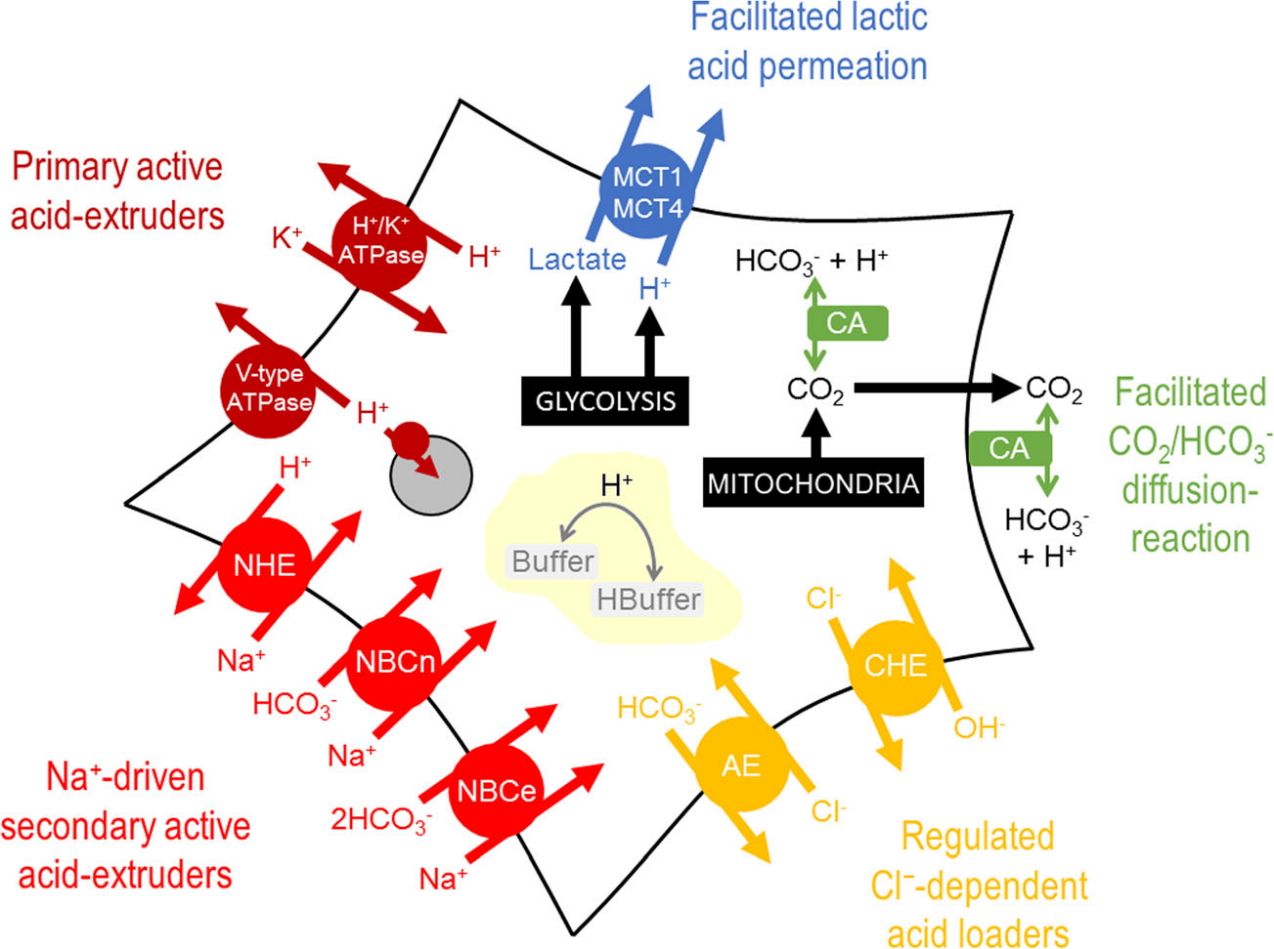
Schematic of a cancer cell, showing the major molecules involved in pH regulation. The complexity of the system is factually correct, but unpalatable for estimating the distribution of H^+^ ions fluxes through the various processes, deriving a value for the steady-state pH_i_, or predicting how the system would respond to changes in one or more of these processes. MCT: H^+^-monocarboxylate transport; CA: carbonic anhydrase; CHE: Cl^−^/OH^−^ exchange; AE: anion exchange; NBCe: electrogenic Na^+^-HCO_3_^−^ cotransport; NBCn: electroneutral Na^+^-HCO_3_^−^ cotransport; NHE: Na^+^/H^+^ exchange; organelle: acidic lysosome/endosome with V-type ATPase

There is now an extensive literature about the various genes and proteins that contribute towards the pH regulatory phenotype of cancer [31, 34–36], producing ever more bewildering schematics such as the one shown in Fig. 1. It falls outside the scope of our intuition to predict, from such schematics, which is the dominant route taken by acid, or how such a system responds to modifications in one or more of its elements (*e.g.*, inhibition by drugs). To fill this niche, mathematical models can be used to simulate complex processes, and arrive at inferences that help in formulating a more accessible narrative. Here, using conceptually simple mathematics (Table 1), I explain the genesis of pH regulation and the role played by the distinct classes of proteins involved in this process.

**Table 1:** List of variables used in the mathematical model for simulating steady-state pH and lactate concentration under the various scenarios presented in Fig.s 2, 3 and 4

Parameter	Definition	Fig 2	Fig 3	Fig 4	*Reference*
*r*	Radius of cell	7 μm	7 μm	7 μm	[37]
*R*	Distance from capillary	(case 1) 0 μm (2–3) 150 μm	(case 1–2) 0 μm (3) 150 μm	150 μm	[38]
pH_i0_	Starting intracellular pH	7.3	7.3	7.3	
pH_ec_	Extracellular pH in capillary	7.4	7.4	7.4	
*β*_int_	Intrinsic buffering capacity	15 mM/pH	15 mM/pH	15 mM/pH	[39]
*β*_e_	Extracellular buffering capacity	3 mM/pH	3 mM/pH	3 mM/pH	[40, 41]
*D*_H_	Interstitial H^+^ diffusion coefficient	12,000 μm^2^/s	12,000 μm^2^/s	12,000 μm^2^/s	[40, 41]
*τ*_e_	Tortuosity in extracellular space	0.5	0.5	0.5	[40, 41]
*J*_CO2_	CO_2_ production rate	0–15 mM/min	0	0	[42]
*K*_CO2_	CO_2_ dissociation constant	10^–6.15^ M	10^–6.15^ M	10^–6.15^ M	
*k*_h_	Spontaneous CO_2_ hydration constant	0.16 s^−1^	0.16 s^−1^	0.16 s^−1^	[38, 40, 41]
CA_e_	Extracellular CA activity	(case 1–2) 1 (3) 1000	1000	1000	[38, 40, 41]
[CO_2_]_ec_	Extracellular CO_2_ concentration in capillary	1.2 mM	1.2 mM	1.2 mM	
*P*_CO2_	CO_2_ membrane permeability	1000 μm^2^/s	1000 μm^2^/s	1000 μm^2^/s	[40]
*D*_CO2_	Interstitial CO_2_ diffusion coefficient	2400 μm^2^/s	2400 μm^2^/s	2400 μm^2^/s	[40]
*D*_HCO3_	Interstitial HCO_3_^−^ diffusion coefficient	1800 μm^2^/s	1800 μm^2^/s	1800 μm^2^/s	[40]
*J*_HLa_	Lactic acid production rate	0	0–20 mM/min	0–20 mM/min	[43–48].
*K*_HLa_	Lactic acid dissociation constant	–	10^–3.86^ M	10^–3.86^ M	
[HLa]_ec_	Extracellular lactic acid concentration in capillary	–	0 mM	0 mM	
*P*_HLa_	Apparent lactic acid membrane permeability	–	(case 1) 10 μm/s (2–3) 1000 μm/s	1000 μm/s	
*D*_La_	Interstitial lactate diffusion coefficient	–	1300 μm^2^/s	1300 μm^2^/s	[40]
*D*_HLa_	Interstitial lactic acid diffusion coefficient	–	1300 μm^2^/s	1300 μm^2^/s	[40]
*V*_max_	Maximum flux generated by active transporter	–	–	(case 1) 0 mM/min (2) 1 mM/min (3–4) 10 mM/min	
*K*_a_	Apparent H^+^ binding constant of active transporter	–	–	(1–3) 10^–7.0^ M (4) 10^–6.7^ M	[31, 39]
*n*	Hill cooperativity of active transporter	–	–	2	[31, 39]
*J*_loading_	Regulated acid-loading flux			(case 1) 0 mM/min (2) 0.2 mM/min (3–4) 2 mM/min	
pH_setpoint_	Intracellular pH setpoint	–	–	(case 1–3) 7.3 (4) 7.0	

## Diffusion and chemical equilibration

**Fig. 2:**
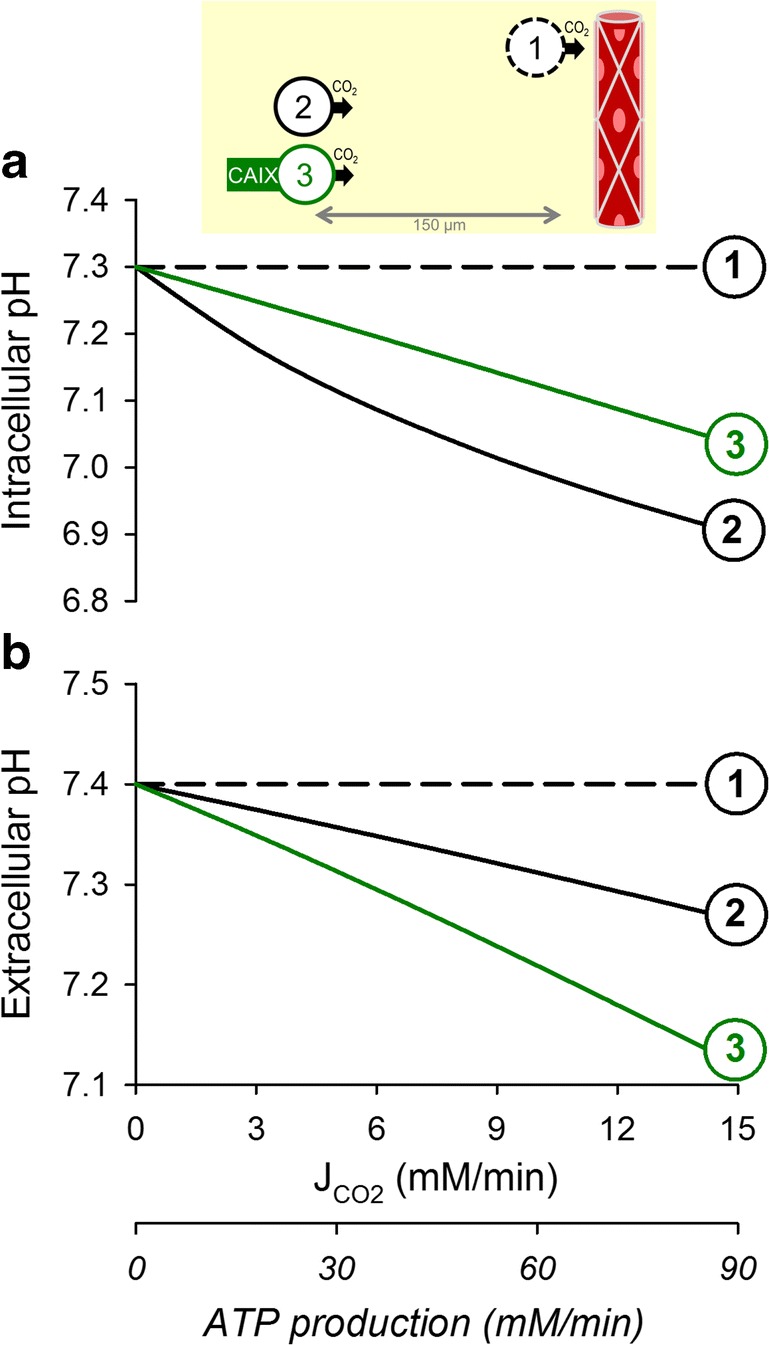
The simulated relationship between aerobically-produced CO_2_ and (**a**) intracellular pH and (**b**) extracellular pH over the range of metabolic rates reported in cells. (1) Cell juxtaposed to a capillary, *i.e.*, absence of a meaningful barrier to CO_2_ diffusion in the extracellular space. Rapid permeation of CO_2_ across the surface membrane results in a tight coupling between intra- and extracellular CO_2_ partial pressures, and hence rapid clearance of CO_2_ from the cell. (2) Distance between the cell and its capillary expanded to 150 μm, a commonly accepted hypoxic limit. This barrier to the flow of CO_2_ produces an intracellular buildup of CO_2_ and establishes a radial gradient of extracellular CO_2_ partial pressure, which is responsible for driving CO_2_ venting. Consequently, both the intra- and extracellular compartments of the tissue acidify. (3) Under adequate CA catalysis (*e.g.* by CAIX), the magnitude of extracellular CO_2_ venting is enhanced by means of facilitated diffusion (transport in the form of HCO_3_^−^ and H^+^ ions), a consequence of which is a further degree of extracellular acidification. More efficient CO_2_ venting reduces the degree of intracellular CO_2_ buildup and intracellular acidification

For the many cells in the body that are juxtaposed to functional capillaries, the supply of oxygen is adequate for aerobic respiration. Such cells, particularly in a differentiated state, would be expected to opt for oxidative phosphorylation as a rich source of ATP [21]. The acidic end-product, CO_2_, is a gas which freely permeates lipid bilayers and possibly also through gas channels [49], although the significance of this facilitated route is debated [40, 50]. CO_2_ production rate can be estimated from measurements of oxygen consumption, which can be as high as 15 mM per minute [42]. Even at these high production rates, biological membranes cannot support gradients of a highly permeant gas, therefore the intra- and extracellular partial pressures of CO_2_ must equalize. Blood capillaries are designed to remove CO_2_ efficiently, and since there are no other barriers to CO_2_ movement, blood perfusion will seamlessly drive CO_2_ out of cells. Under these circumstances, pH_i_ remains constant, as there is no meaningful buildup of CO_2_ (Fig. 2a(1)). For the simulations shown in Fig. 2, starting pH_i_ was set at 7.3, the predicted optimal for cytoplasmic enzymes. Whilst efficient CO_2_ removal ensures the constancy of pH_i_, it cannot influence the level at which pH_i_ is kept. Offsetting pH_i_ relative to pH_e_ ultimately requires an input of energy, whereas the process of CO_2_ venting is purely dissipative.

In poorly perfused tissues, such as tumors, the distance to the nearest capillary can become substantial. This constitutes a barrier to CO_2_ movement, which requires an adequately steep gradient of CO_2_ partial pressure to drive the flow of gas: invariably, cells will accumulate CO_2_ and acidify (Fig. 2a(2)). This scenario also leads to an undesirable coupling between pH_i_, diffusion distance, and metabolic rate, which greatly limits the scope of cancer cell behaviors. A way of improving CO_2_ venting is to increase its effective diffusivity by enabling a parallel transport of H^+^ and HCO_3_^−^ ions. The necessary chemical conversion is normally very slow, but can be catalyzed enzymatically by exofacial isoforms of carbonic anhydrase, such as CAIX and CAXII (coded by genes *CA9*, *CA12*) [38, 51–53]. Faster CO_2_ clearance reduces the extent of intracellular acidification (Fig. 2a(3)), but also leads to a more pronounced extracellular acidification (Fig. 2b(3)). This latter effect has been documented in 3D spheroids of cancer cells *in vitro* [54] and in xenografts *in vivo* [55], and is believed to be important in the acid-selection process in cancer [6, 22].

## Facilitated membrane permeation

**Fig. 3:**
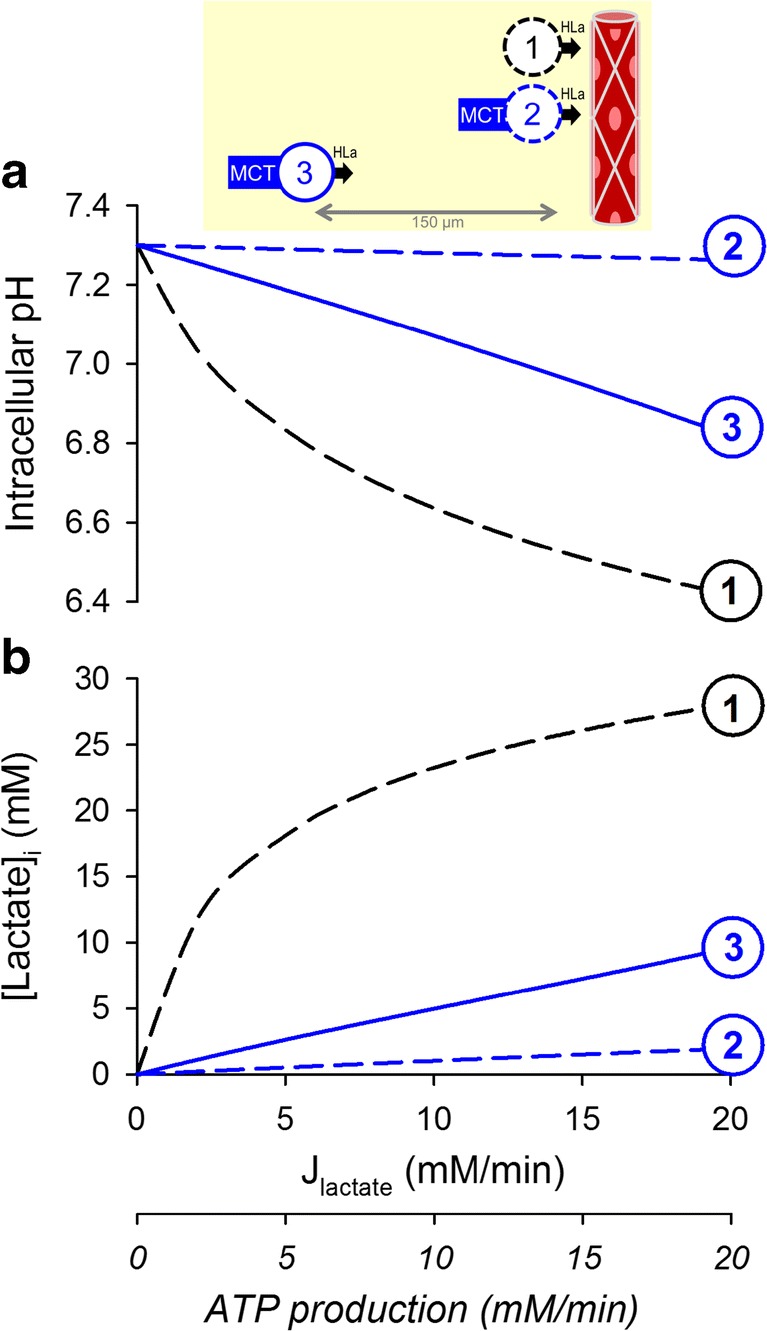
The simulated relationship between glycolytic lactic acid production and (**a**) intracellular pH and (**b**) intracellular lactate retention over the range of metabolic rates reported in cancer cells. (1) Cell lacking protein-facilitated permeability to lactic acid, juxtaposed to a capillary, *i.e.*, absence of a meaningful barrier to lactic acid diffusion in the extracellular space. Since lactic acid is only poorly permeant across lipid bilayers, its venting is severely restricted by the cell membrane, resulting in an intracellular buildup of lactate and H^+^ ions. (2) Cell with high lactic acid permeability attained with MCT isoforms (*e.g.*, MCT1 and MCT4), juxtaposed to a capillary. With higher permeability, a much smaller gradient is required to drive lactic acid efflux, resulting in a considerably diminished intracellular buildup of lactate and H^+^ ions. (3) Distance between the cell and its capillary expanded to 150 μm, a commonly accepted hypoxic limit. CO_2_/HCO_3_^−^ equilibration is ensured by high CA activity. As a consequence of the extracellular diffusional barrier to lactic acid movement, a substantial gradient of lactic acid is required to drive venting. This results in a greater intracellular retention of H^+^ and lactate ions. Thus, steady-state pH_i_ becomes subservient to both metabolic rate and distance from capillary, *i.e.*, is not independently regulated

In underperfused tissues, the diffusion path that restricts the outflow of CO_2_ will also restrict the counterflux of oxygen. With reduced O_2_ penetration, tumor cells must rely on glycolysis. Intriguingly, cancer cells typically manifest a glycolytic phenotype even in the presence of oxygen, a phenomenon known as the Warburg Effect [56]. The rates of lactic acid production by cancer cells are in the low mM/min range [43–47], but some cancer cells can attain rates as high as 20 mM/min [48]. Compared to CO_2_, lactic acid ionizes more completely, which reduces the availability of its uncharged lipid-soluble form. Thus, lactic acid permeability across lipid bilayers is low in relation to the venting demand placed by glycolysis. Without any form of facilitated permeation, a substantial transmembrane gradient of lactic acid would be necessary to drive an adequate efflux: consequently, cells would accumulate high levels of lactic acid and lactate (Fig. 3b(1)). A solution to this conundrum is in the form of H^+^-monocarboxylate transporters of the *SLC16* gene family [57], such as the ubiquitously expressed MCT1 (*SLC16A1*). By shuttling H^+^ and lactate ions across membranes, MCTs greatly increase the apparent membrane permeability to lactic acid; consequently, a much smaller concentration gradient is necessary to drive an adequate lactic acid efflux (Fig. 3a/b(2)). In the case of well-perfused cells expressing MCT isoforms, intracellular lactate accumulation and acidification are minimal and compatible with pH_i_ constancy. However, this system is unable to offset pH_i_ to a desired set point because protein-assisted permeation is solely dissipative.

In under-perfused tumors, the diffusion distance across the interstitium is an additional “resistance” to the flow of lactic acid, which mostly takes the form of lactate and H^+^ ions. Cells in such niches may induce hypoxia-upregulated MCT4 to minimize the permeability barrier at their surface membrane [58], but this response cannot address the problem of diffusion across the interstitial space. Of the two chemical species released by glycolytic cells, the diffusive flux of H^+^ ions is likely to be rate-limiting because it is dramatically restricted by reversible binding to buffers [59–61] in an environment that does not support fast transport involving proton wires (Grotthuss mechanism) [62]. H^+^ ion diffusion can be facilitated by the mobile CO_2_/HCO_3_^−^ buffer with adequate levels of exofacial CA activity; however, even with maximal enzymatic facilitation, the diffusional barrier cannot be collapsed. In glycolytic tissues, the diffusional delay across the interstitium will result in an intracellular retention of lactate and H^+^ ions, reaching levels that may become physiologically untenable (Fig. 3a/b(3)). These circumstances would justify the implementation of additional homeostatic measures, ultimately resorting to uphill (active) transport.

## Active transport and the pH set point

The components of pH regulation described thus far address the issue of slow diffusion of the CO_2_/HCO_3_^−^/H^+^ system across extracellular spaces (CA) and inadequate lactic acid permeation across membranes (MCT). These protein-assisted processes are passive: they do not consume energy but, instead, hasten equilibration. It would be thermodynamically implausible for these processes, alone, to maintain tumor pH_i_ at a certain set point under continuous metabolic acid loading. Any departure from the “passive” pH_i_ and pH_e_ curves plotted in Figs 2 and 3 would require a form of active transport, which historically has been at the center of research into pH regulation. There are many types of transporters that engage in active transport, and these can be classified as being either primary active (V-type H^+^ ATPase, P-type H^+^/K^+^ ATPase) or secondary active (*e.g.*, Na^+^/H^+^ exchangers of the *SLC9* gene family [63]) [31, 34–36]. The latter class also includes transporters that carry HCO_3_^−^ ions, which is chemically equivalent to a counterflux of H^+^ ions (*e.g.*, Na^+^-HCO_3_^−^ cotransporters of the *SLC4* gene family, see Bødtkjer, this volume) [64]. Whilst HCO_3_^−^-importing pH_i_ regulators can be distinguished from H^+^-exporting counterparts by experimental maneuvers (*e.g.*, the system’s response to the removal of CO_2_/HCO_3_^−^ buffer) [65], their physiological outcomes are equivalent: both produce an equimolar intracellular alkalinization.

In homeostatic terms, a more relevant characterization of pH_i_-regulating proteins relates to their kinetics, rather than the chemical identity of the transport substrate. The maximal transport rate (*V*_*max*_) describes the capacity for surface-expressed transporters to produce a flux of H^+^ ions or their chemical equivalents. A powerful pH_i_-regulatory system is expected to produce fluxes that comfortably exceed the sum of disturbances, such as glycolysis. However, for such a system to be efficient, its energetic footprint must not be excessive to avoid an unwarranted depletion of ATP. pH_i_ regulators must also receive feedback that gauges the progress of their actions: as pH_i_ rises, the acid-extrusion process should slow. The relationship between flux and pH_i_ can be described in terms of an apparent affinity constant (*K*_a_) and cooperativity (a measure of steepness). Although high pH_i_ can allosterically inhibit acid-extrusion, it cannot block this efflux completely within the physiological pH_i_ range. Consequently, a regulatory system comprising only of acid-extruders would manifest an upwardly drifting pH_i_ rather than stabilize at a steady-state pH_i_. To ensure that the steady-state condition is met, acid-extrusion at the desired set point pH_i_ must be matched by an equal acid-loading flux, such as that generated by the activity of various Cl^−^-coupled transporters belonging to the *SLC4* or *SLC26* families of genes [66–68]. The magnitude of these equal but opposite acid-fluxes determines the robustness of the system’s response to acid-base disturbances, in addition to its baseline energy consumption. For example, higher fluxes make the system better at defending pH_i_ during transient challenges, such as bursts of metabolic activity, but these require higher ATP production to power the apparently futile cycle of Na^+^-dependent acid-extrusion and Cl^−^-dependent acid-loading. The compromise that a cell strikes between these conflicting interests influences its survival in acidic niches.

**Fig. 4:**
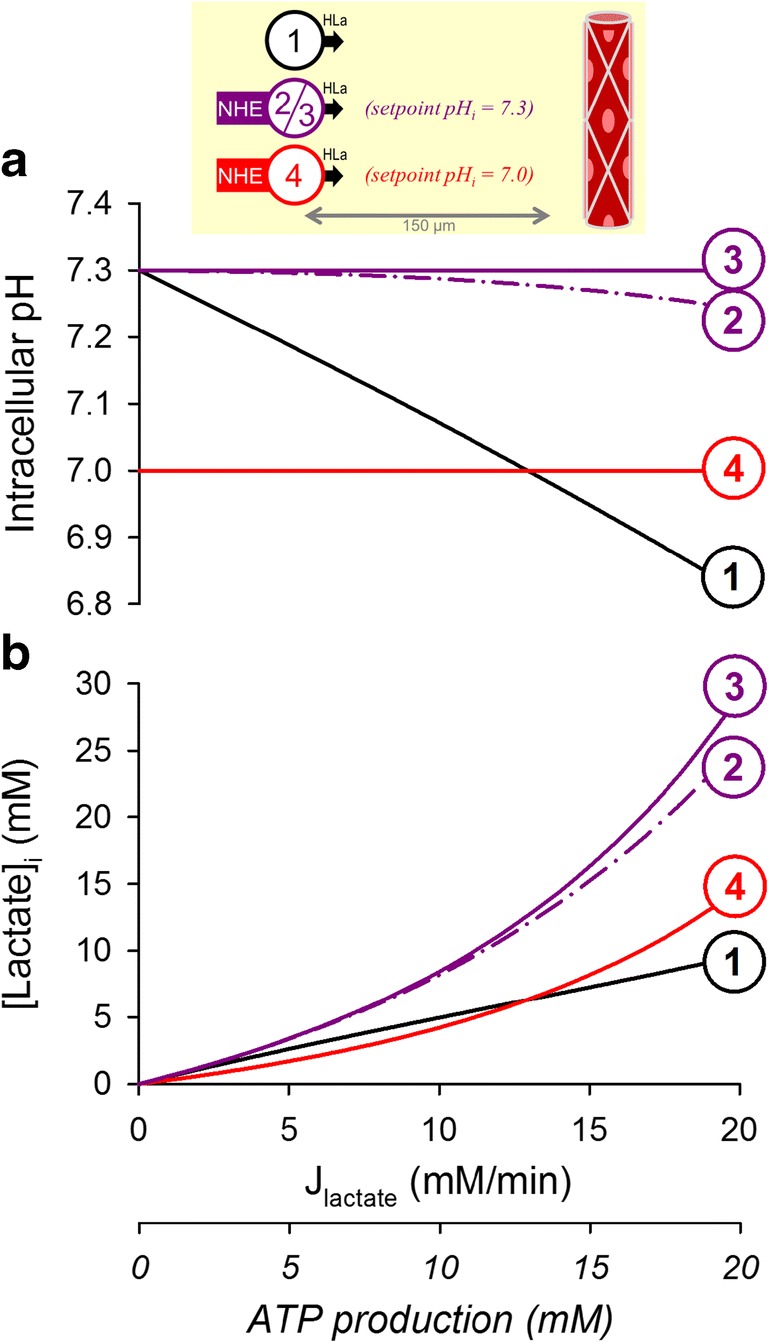
Simulating the effect of active transport on the relationship between glycolytic lactic acid production and (**a**) intracellular pH and (**b**) intracellular lactate retention. (1) Cell with high exofacial CA activity and high lactic acid permeability placed 150 μm away from a capillary. (2) Inclusion of an active transporter, such as Na^+^/H^+^ exchanger, with a set point at pH_i_ = 7.3 and maximal acid extrusion rate of 1 mM/min; this relatively low corrective flux is unable to fully offset metabolic acid-loading, resulting in a modest influence of glycolytic rate on pH_i_. Uphill extrusion of H^+^ ions from the cell favors lactic acid dissociation and increases intracellular lactate retention. Active transport will reduce net ATP supply by 0.07 mM/min. (3) Raising the maximal acid-extrusion flux by 10-fold is sufficient to maintain pH_i_ at the set point of 7.3 over a wide range of metabolic rates; this establishes a system that truly regulates pH_i_, independently of constraints imposed by metabolic rate or diffusion distance. However, clamping pH_i_ to 7.3 results in substantial intracellular lactate retention. Additionally, the elevated rate of active transport reduces ATP supply by 0.7 mM/min. (4) Lowering the set point of active transport from 7.3 to 7.0 reduces the degree of lactate retention inside cells, whilst still defending constancy of pH_i_, albeit at a less alkaline level

To explore how the various parameters relating to active transport influence steady-state pH_i_, a simplified kinetic representation of acid-extrusion, designed to defend a set point pH_i_ of 7.3, was included in the model. The transporter’s pH_i_-sensitivity was modeled with a pK_a_ that was 0.3 units lower than the set point pH_i_, and a cooperativity of 2. These values are within the range reported for Na^+^/H^+^ exchangers expressed in cancer cells [31, 37, 39]. For a maximal flux (*V*_max_) set to 1 mM/min, the balancing acid-loading flux would need to be 0.2 mM/min, *i.e.*, an ATP consumption of 0.07 mM/min (calculated on the basis that the Na^+^/K^+^ pump which ultimately drives secondary-active transport has a stoichiometry of 3Na^+^/ATP). This relatively low flux is inadequate to defend pH_i_ in highly glycolytic and diffusively-restricted tumors (Fig. 4a(2)). Raising *V*_max_ to 10 mM/min produces a system that is able to maintain pH_i_ at the set point, even under high glycolytic rates, but its higher ATP demand (0.7 mM/min) is the price the cell must pay for the improvement in pH_i_ control (Fig. 4a(3)).

**Fig. 5:**
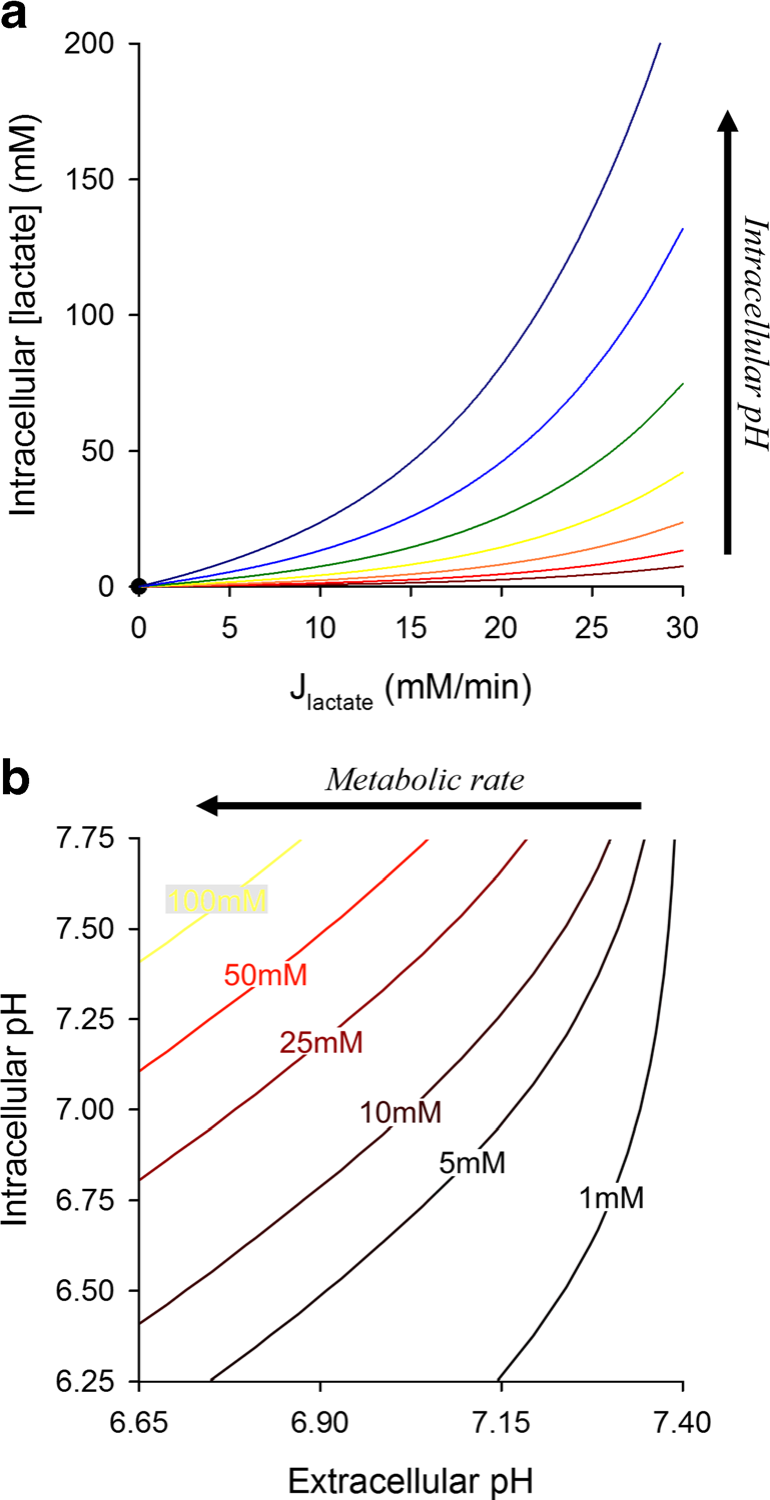
Using the mathematical model to map the relationship between pH_i_, pH_e_, and intracellular lactate. Simulations were based on the model for a glycolytic cells with high MCT and exofacial CA activity, placed 150 μm away from a capillary. pH_i_ and lactic acid production were varied between 6.25 and 7.75, and 0 and 30 mM/min, respectively. (**a**) Intracellular lactate concentration as a function of metabolic lactate production rate; each line represents a different starting pH_i_. (**b**) Results replotted as a contour map. Contours show the combination of pH_i_ and pH_e_ that yield a particular concentration of lactate in cytoplasm. The highest degree of intracellular lactate retention is attained with high metabolic rates, when the inward pH gradient is large (*i.e.*, pH_i_>pH_e_)

A consequence of regulating pH_i_ to an alkaline set point is that it produces a cytoplasmic milieu that favors lactic acid dissociation. Cells in diffusively-restricted tissues will thus build up lactate to levels that can be significant, reaching tens of mM, and likely exerting functional consequences, such as end-product inhibition of glycolysis. Thus, it may not necessarily be desirable for glycolytic tumors to maintain their pH_i_ much higher than 7.0 because this invariably leads to intracellular lactate retention. Since the transmembrane distribution of lactate is set by the pH_i_/pH_e_ gradient, one way of “regulating” lactate is by adjusting set point pH_i_ towards a less alkaline level; for example, dropping this from 7.3 to 7.0 halves lactate retention (Fig. 4a(4)) without altering ATP consumption (assuming that the regulated acid-loading flux is of the same magnitude at the new steady-state pH_i_). To explore this further, simulations were run for a range of starting pH_i_ and metabolic rates (Fig. 5a). The concentration of intracellular lactate attained under the simulated conditions is shown by the contour plots in Fig. 5b, and demonstrates why maintaining an invariably alkaline pH_i_ in a milieu of low pH_e_ may become disadvantageous for glycolytically-active tumors. Indeed, it is well-documented that even in well-perfused single cells, steady-state pH_i_ falls modestly in response to a decrease in pH_e_, producing a coupling between pH_e_ and pH_i_. A reason behind this seemingly imperfect homeostatic apparatus may be to strategically protect cells from excessive lactate retention, which would otherwise happen if pH_i_ remained  substantially higher than pH_e_. Thus, the burden of lactate retention is lessened by allowing cells to modestly acidify in niches of low pH_e_.

## Predicting a cell’s steady state pH

**Fig. 6:**
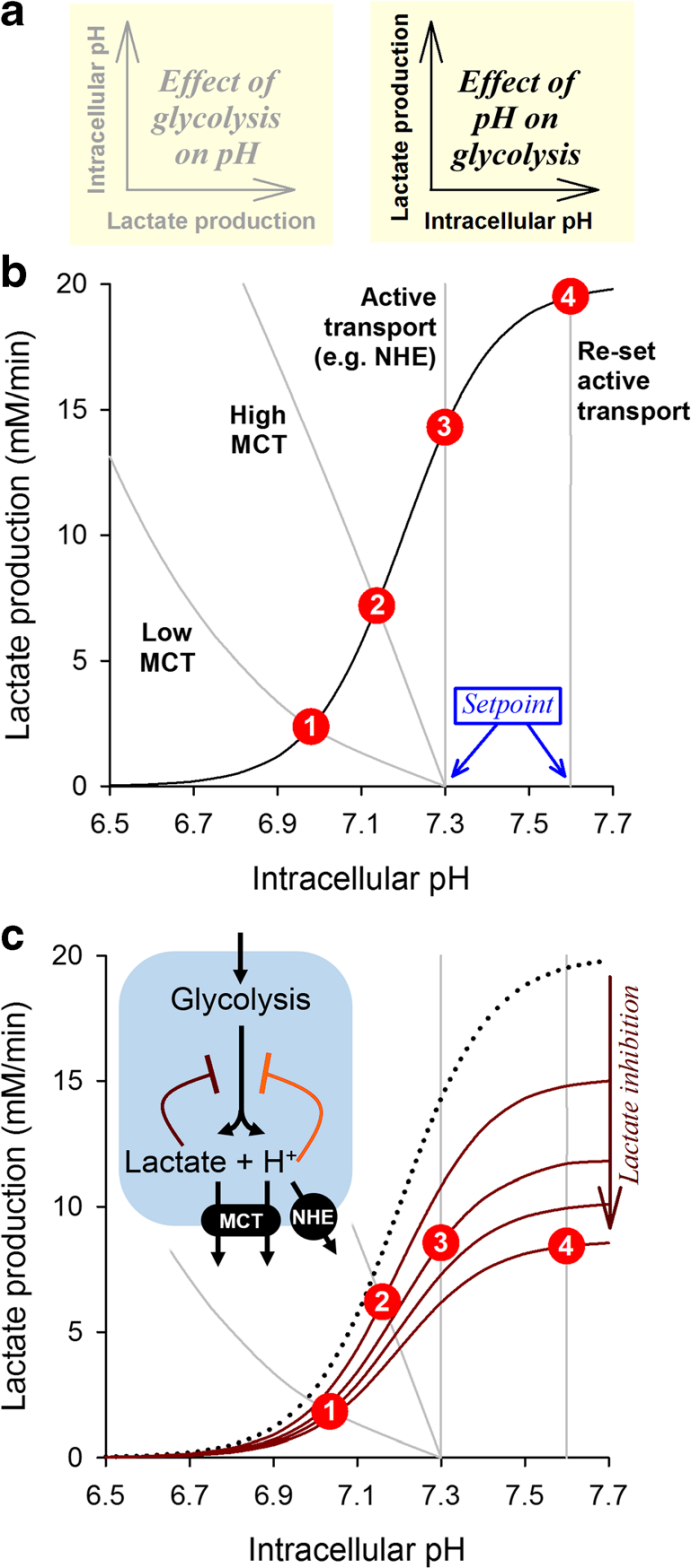
Using a graphical approach to infer steady-state pH_i_ and metabolic rate. **(a)***Left:* Lactate production affects intracellular pH, as described in Fig. 3 and Fig. 4. In this representation, metabolic rate is considered to be the independent variable. *Right*: Intracellular pH affects lactate production through the inhibition of glycolysis. In this representation, pH_i_ is considered to be the independent variable. **(b)** The pH_i_-metabolism and metabolism-pH_i_ relationships are akin to two equations; the mathematical solution to these can be inferred graphically from the point of crossover. Superimposing the relationship between pH_i_ and glycolytic rate (black curve) with the relationship describing the effect of glycolytic rate on pH_i_ (gray curve) for a cell, located 150 μm away from its nearest capillary, with high CA expression and either (1) low MCT activity, (2) high MCT activity, (3) high MCT activity and active transport with a set point of pH=7.3, or (4) high MCT activity and active transport with a set point of pH=7.6. The points of crossover (red ;circles) are the mathematical solutions of these four pairs of equations. Steady-state pH_i_ and metabolic rate increase in tandem when MCT activity is raised and when active transport is engaged to an alkaline set point. **(c)** In addition to the allosteric inhibitory effect of H^+^ ions on glycolytic enzymes, another influence is end-product inhibition of glycolysis by the accumulation of lactate. This is expected to scale-down the pH_i_-metabolism curve and produce a different crossover point, particularly at high pH_i_ when lactate accumulation is expected to be substantial. In the example illustrated (assuming an inhibitory constant K_i_ of 10 mM for lactate), the inhibitory effect produced by intracellular lactate retention at pH_i_ > 7.3 offsets the disinhibition of glycolysis by low [H^+^]; consequently, the highest possible metabolic rate is attained in the range 7.1–7.3

The discussion of pH regulation so far has focused on how metabolic acid production influences steady-state pH in the intra- and extracellular compartments of tissue (Fig. 6a(left)). In parallel, pH feeds back on metabolic rate through the inhibitory effect of intracellular H^+^ ions on glycolytic enzymes (Fig. 6a(right)) [21]. For example, phosphofructokinase, the enzyme catalyzing the rate-limiting step of glycolysis, manifests a steep pH-sensitivity. The relationship between pH_i_ and glycolysis can be modeled with a curve such as that shown in Fig. 6b. The pH_i_-metabolism relationship (where pH_i_ is the independent variable) and the inverse metabolism-pH_i_ relationship (where metabolic rate is the independent variable) can be superimposed to obtain the mathematical solution describing steady state pH_i_ and metabolic rate. This can be visualized as the point of crossover of the two relationships. Increasing MCT activity (in the absence of active transport) allows pH_i_ and metabolic rate to increase in tandem (Fig. 6b: 1 to 2). A further up-lift is attained by incorporating active transport (Fig. 6b: 2 to 3), and even more so if the transporter is adjusted to a higher set point pH (Fig. 6b: 3 to 4). This simplified analysis can be helpful in explaining the dynamic interplay between metabolism and pH.

Given that metabolism is a limiting factor for cancer cell proliferation, it would seem desirable for tumors to express high levels of MCT and to offset pH_i_ to an alkaline level by active transport. However, the metabolic rate plotted in Fig. 6b does not consider the effect of intracellular lactate accumulation (*cf.* Fig 5), which could exert end-product inhibition on glycolysis [21]. Because this thermodynamic consequence is not inherently cooperative, its effect on metabolic rate is expected to be smaller than the allosteric inhibition of enzymes by H^+^ ions. However, at profoundly alkaline pH_i_, the allosteric disinhibition of glycolytic enzymes plateaus and the inhibitory effect of lactate accumulation becomes overriding. This effect of lactate can be modeled as a down-scaling of the pH_i_-metabolic rate curve, as shown in Fig. 6c. A somewhat surprising consequence of the dual inhibition by lactate and H^+^ ions is that a profoundly alkaline cytoplasm may not necessarily be conducive for a high metabolic rate, because the inhibitory effect of lactate retention may cancel-out the benefit of enzyme disinhibition at low [H^+^]. This interaction may explain why most tumors have a pH_i_ in the mildly alkaline range, around 7.2 [16, 69]: a tested compromise between a pH_i_ that is sufficiently alkaline to disinhibit glycolysis but not at a level that would overload the cytoplasm with lactate anions.

## Conclusions

Since the milestone discoveries of cellular pH regulation by Roger Thomas, Walter Boron, Richard Vaughan-Jones, Andrew Halestrap, and many others, our understanding of acid-base homeostasis has developed to a fine level of molecular detail thanks to breakthroughs in physiology, molecular biology, and genetics. Complex systems, like pH regulation, are not intuitive to understand, and can be misinterpreted if our analytical framework is not adequately integrative, *i.e.*, when it considers a subset of components of the system in isolation. Although therapeutic interventions aimed at disturbing pH regulation are typically targeted to meet the criteria for clinical translation, their effects on pH_i_ and pH_e_ will be highly context-sensitive, and depend on factors such as metabolic rate, diffusion distances, and the repertoire of other pH-regulating molecules. This problem highlights the need to characterize pH regulation in as much detail as possible, and use calibrated mathematical models to identify a suitable Achilles heel for targeted disruption. To make such models accurate yet accessible, they must be simple to understand and supply with parameters, but not any simpler (Albert Einstein, 1950).

The analyses shown in this review are based on representative parameters obtained from the literature and must not be generalized to all cases of tumors; rather, the graphical illustrations should be used a didactic guides for explaining the scope of various elements of pH regulation in influencing pH and lactate concentration. The modeling scenarios discussed herein assume that cells behave as independent units in terms of pH_i_ regulation. Most cells in the body are, however, diffusively coupled by means of channels, such gap junctions formed by connexins. Such coupling would result in syncytial behaviors of clusters of cells, but the relevance of this to cancer is likely to be limited to special cases, because gap junctional coupling tends to be low or absent in tumors [70], possibly due to the tumor-suppressing effect that has been attributed to connexins [71, 72]. Nonetheless, there are cases of well-coupled cancer cells, and in such instances, pH regulation would operate in a syncytial mode [73, 74].

Some key points borne from the analyses presented herein are paraphrased below:

1. CO_2_ permeation across membranes is fast and unlikely to be a substantial barrier to CO_2_ movement. Consequently, no significant gradients in CO_2_ partial pressure are expected between cells and their immediate microenvironment.
2. Interstitial diffusion distances in poorly-perfused tissues can impose a meaningful resistance to CO_2_ movement. CO_2_ diffusion can be facilitated by a parallel flux of HCO_3_^−^ and H^+^ ions, but only in the presence of extracellular carbonic anhydrase (CA) activity. This CA-catalyzed CO_2_ clearance will alkalinize cytoplasm and acidify extracellular spaces.
3. In contrast to CO_2_, lactic acid crosses lipid bilayers very slowly and therefore its permeation must be assisted by H^+^-monocarboxylate co-transporters (MCT); otherwise, lactic acid and lactate will accumulate intracellularly to untenable levels, even in well-perfused cells.
4. Lactic acid diffusion across the interstitium is a resistance in series with membrane permeation, and therefore cannot be augmented by MCT expression at the cell surface. Since lactic acid almost fully ionizes, a rate-limiting step to its venting is likely to be the diffusion of H^+^ ions, which is greatly restricted in biological fluids. This limiting step can be assisted by CO_2_/HCO_3_^−^ buffer, which acts as a mobile H^+^ shuttle, if there is adequate extracellular CA activity.
5. Overall, exofacial CA isoforms improve acid venting from cells by facilitating diffusion. However, this beneficial effect will only be meaningful in the context of long diffusion distances. Thus, it is not possible to demonstrate a meaningful CA-related effect on pH_i_ regulation in isolated cells or well-stirred monolayers, where extracellular diffusion distances are negligible.
6. Cells that express extracellular-facing CA isoforms and MCT at their membrane improve their bandwidth for venting acidic end-products, but their pH_i_ will become subservient to metabolic rate and diffusion distance in a manner that does not meet the strict criteria for true pH_i_ homeostasis. These criteria are met by the inclusion of active transporters that generate uphill movement of H^+^ ions (or their chemical equivalents; *e.g.*, HCO_3_^−^) across membranes. Active transport can thus uncouple pH_i_ and pH_e_ from the constraints of passive equilibration.
7. Active transporters will produce a meaningful correction to pH_i_ if the H^+^/H^+^-equivalent flux they generate is adequately high. The magnitude of this flux depends on maximal turnover and allosteric modulation by H^+^ ions. For typical metabolic rates, fluxes greater than several mM/min are necessary for the pH_i_ regulatory system to achieve adequate homeostatic power.
8. Given that acid-loading by metabolism is the primary threat to pH housekeeping, it may seem counterproductive for cells to express acid-loading transporters at the membrane. However, these regulated acid-loading fluxes are mandated for balancing acid-extrusion and stabilizing pH_i_ at a particular level.
9. The energy consumed by acid-extruding active transporters relates to the magnitude of the regulatory acid-loading fluxes that must work against them. The ATP-cost of this balancing act places a limit on how responsive a cell’s pH_i_ regulatory system can become. Typical ATP consumption rates are in the high μM/min to low mM/min range.
10. Various enzyme-catalyzed processes can be ascribed specific pH_i_-optima; glycolytic rate is, overall, faster at higher pH_i_. However, underperfused glycolytic tissues may not necessarily find it beneficial to maintain an alkaline pH_i_ because this leads to a greater retention of lactate in cytoplasm, which itself may exert end-product inhibition on glycolysis. This reasoning may explain why the cell’s set point pH_i_ tends to decrease at low pH_e_: a pre-emptive action to limit the degree of lactate accumulation.
